# Illumination-Based Synchronization of High-Speed Vision Sensors

**DOI:** 10.3390/s100605530

**Published:** 2010-06-02

**Authors:** Lei Hou, Shingo Kagami, Koichi Hashimoto

**Affiliations:** Department of System Information Sciences, Graduate School of Information Sciences, Tohoku University, 6-6-01 Aramaki Aza Aoba, Aoba-ku, Sendai 980-8579, Japan; E-Mails: swk@ic.is.tohoku.ac.jp(S.K.); koichi@ic.is.tohoku.ac.jp(K.H.)

**Keywords:** camera synchronization, phase-locked loop, visible light communication

## Abstract

To acquire images of dynamic scenes from multiple points of view simultaneously, the acquisition time of vision sensors should be synchronized. This paper describes an illumination-based synchronization method derived from the phase-locked loop (PLL) algorithm. Incident light to a vision sensor from an intensity-modulated illumination source serves as the reference signal for synchronization. Analog and digital computation within the vision sensor forms a PLL to regulate the output signal, which corresponds to the vision frame timing, to be synchronized with the reference. Simulated and experimental results show that a 1,000 Hz frame rate vision sensor was successfully synchronized with 32 *μ*s jitters.

## 1. Introduction

When multiple vision sensors are used to acquire images of a scene from multiple points of view to achieve, for example, cooperative tracking, wide area monitoring or 3D motion measurement, the image sequences given by the sensors should be synchronized. Synchronization of image sequences in general involves two concepts: One is to produce temporally-aligned vision frames in image acquisition, and the other is to establish correct correspondence between the vision frames. The former must be done just during the image acquisition phase, while the latter can be done on-line, off-line, or in combination of the both. This paper is concerned with the former, and aims at proposing a novel synchronization technique that can be used even for low-cost wireless vision sensor networks. The term “synchronization” is used in this former sense throughout this paper unless otherwise stated.

Synchronization of vision sensors is a critical demand in some applications in the fields of, in particular, industrial or scientific measurement. In some applications, virtual synchronization, e.g., [1], in which interpolation and prediction between the frames of unsynchronized cameras are used, can be an alternative, but real synchronization is apparently advantageous when, for example, the motion of target objects is fast and random, and/or highly precise position information is required.

Thus many of industrial vision sensors are equipped with dedicated electrical inputs/outputs for synchronization trigger signals, in which one of the vision sensors—or a dedicated signal emitter device—acts as a master, and the others are operated in synchronization with the trigger signal emitted from the master. A major problem in this classical and widely-used means is that deployment of synchronization wires is cumbersome in some situations—short wires may impose constraints on spatial configuration of vision sensors; long wires may cause unstable synchronization.

Instead of dedicated synchronization wires, some systems allow synchronization through standard electronic buses used for image transfer such as IEEE 1394 [2] and Ethernet [3,4]. These systems bring higher flexibility, but they still require wired connections and are unsuitable for wireless vision sensor networks.

The principal difficulty in time synchronization of wireless network systems lies in nondeterminism in wireless media access time [5]. Due to this nondeterminism, it is difficult to make certain when a synchronization packet started to propagate from the sender. RBS [6] introduced a receiver-receiver synchronization scheme to remove the effect of the sender nondeterminism, but requires many message exchanges between receivers to achieve high precision. TPSN [7] and FTSP [8] suppress this nondeterminism by timestamping at the Media Access Control (MAC) layer, but they inherently require special MAC implementations.

In this paper, we explore into an illumination-based synchronization for vision sensors. Illumination source is always needed in visual sensing unless the observed target itself is a light emitter. In the proposed method, we introduce an intensity-modulated light source as the illumination, where the modulation frequency is set to a half of the desired vision frame rate. On the vision sensor side, we require no dedicated receivers, but the imager itself serves as the receiver of the synchronization signal. Actually, the proposed method do not require any communication media other than the visual information, and thus is applicable even to vision sensors equipped with no communication capability but with only data storage for image or image-feature sequences.

**Figure 1.**
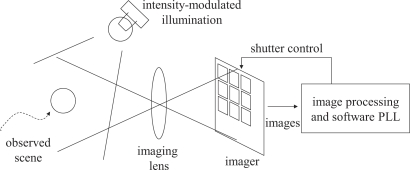
Conceptual diagram of the proposed illumination-based synchronization.

The proposed algorithm is based on the Phase-Locked Loop (PLL) technology [9,10]. Figure 1 illustrates the conceptual diagram of the proposed system. Incident light to the vision sensor serves as the reference signal for synchronization. Internal functions of the vision sensor, including the analog photo integration process in the imager and digital computation executed outside of the imager, forms a PLL to regulate the output signal, which corresponds to the vision frame timing, so that the output is synchronized with the reference.

Because the modulation frequency of the illumination must be a half of the desired vision frame rate, blinking of the illumination may be visible to human eyes depending on the desired vision frame rate. For example, when the desired frame rate is 30 Hz, the illumination should be modulated at 15 Hz and will irritate human eyes. This will be problematic, for example, when measurement is carried out in human-coexisting environments and also when the measured target is human.

One way to address this issue is to use an invisible light such as infrared light just like many industrial visual measurement systems do, and another way is to focus on high frame rate cases in which the modulation effect of illumination is not perceptible to human eyes at all. The description in this paper—particularly that in the simulations and experiments sections—focuses on the high frame rate cases because those cases are more challenging in terms of synchronization stability, but the application scope of the proposed method is not limited to them.

This paper is a revised and extended version of our conference paper [11] with more rigorous descriptions of the theory, particularly in explaining the effect of shorter exposure time than the frame time, and more detailed simulated evaluation. The remainder of the paper is organized as follows: in Section 2 the synchronization algorithm is presented. Section 3 introduces the MATLAB simulation and the analysis of the behavior of the algorithm. In Section 4, experimental results are presented and the proposed method is evaluated. Finally, conclusions are drawn in Section 5.

## 2. Synchronization Algorithm

### 2.1. PLL Theory

For better understanding of the proposed method, we first describe an overview of the PLL theory. A PLL is a system synchronizing an output signal with a reference or input signal in frequency as well as in phase. In particular, our proposal is based on the binary-valued continuous-time PLL, where the reference and output signals are square waves.

**Figure 2.**
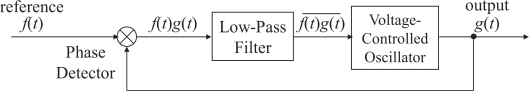
Block diagram of a PLL.

**Figure 3.**
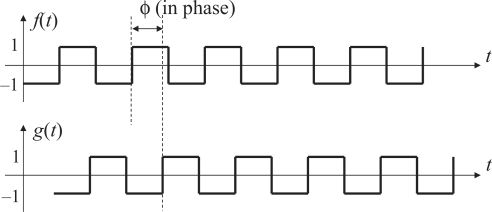
The reference signal f(t) and the output signal g(t).

**Figure 4.**
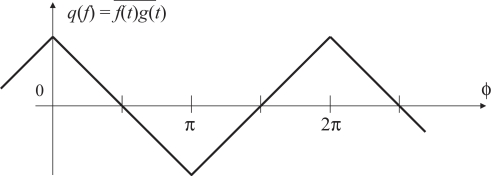
The relation between the time correlation and the phase difference.

In Figure 2, which illustrates a block diagram of a typical PLL, the product *f*(*t*)*g*(*t*) of the reference signal *f*(*t*) and the output signal *g*(*t*) is computed by the phase detector. As shown in Figure 3, both of *f*(*t*) and *g*(*t*) are square waves that alternately take two values, namely 1 and −1, with 50 % duty ratio. The product *f*(*t*)*g*(*t*) is filtered by a low-pass filter to yield an averaged value $\bar{f(t)g(t)}$ of the product. The averaged product $\bar{f(t)g(t)}$, which is also known as the time correlation of the signals *f*(*t*) and *g*(*t*), depends on the phase difference *ϕ* between *f*(*t*) and *g*(*t*). The relation between $q(\phi )\equiv \bar{f(t)g(t)}$ and *ϕ* is shown in Figure 4.

The time correlation is taken by a voltage-controlled oscillator (VCO) whose output frequency varies depending on its input *q*(*ϕ*). When *q*(*ϕ*) equals to zero, it oscillates at a predetermined central frequency. The larger *q*(*ϕ*) is, the lower the output frequency is; the smaller *q*(*ϕ*) is, the higher the frequency is. A typical design is to make the output frequency linearly depend on the input voltage *q*(*ϕ*), but saturate at certain lowest and highest input values.

As can be seen from Figure 4, when *q*(*ϕ*) is positive, the output signal *g*(*t*) is varied in the direction such that its phase will be lagged relatively from that of the reference *f*(*t*). When negative, of course, *g*(*t*) is varied so that its phase is advanced. As long as the feedback characteristic of this loop is properly designed, the system will be converged to the stable equilibrium point *q*(*ϕ*) = *π/*2. This is called the locked state. Note that the point *q*(*ϕ*) = 3*π/*2 is an unstable equilibrium point, and thus the system will not stay here in a real operation environment where various disturbances exist.

### 2.2. Imager-based PLL – A Simplified Case

In the proposed synchronization system, we take the sum of the incident light power accepted by all the pixels of the imager of the vision system as the reference signal *f*(*t*). For the sake of simplicity, we assume, for the moment, that there is no background light, that is, all of the light component that the imager accepts is originated from the modulated illumination. We also assume that the average brightness of the scene does not change too rapidly and the amplitude of *f*(*t*) is considered to be constant, even when some of the pixels are saturated. Then, without loss of generality, *f*(*t*) can be defined by a square wave with the low value 0 and high value 1. The final assumption we make for the moment is that the photo integration time of the vision sensor is equal to its frame time. Loosening or validating these assumptions will be discussed later.

**Figure 5.**
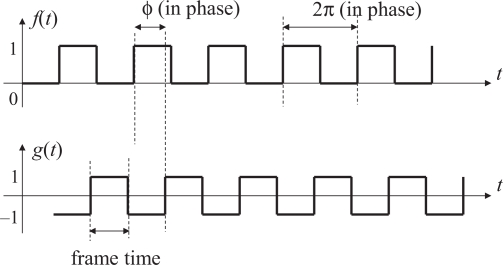
Signals f(t) and g(t) defined in the proposed synchronization system.

We also define, as the output signal of the PLL, a time function *g*(*t*) such that *g*(*t*) = 1 when the frame number index of the vision sensor is odd and *g*(*t*) = −1 when even. The signals *f*(*t*) and *g*(*t*) are illustrated in Figure 5. Note that the vision frame rate will be exactly twice the illumination modulation frequency when the PLL system is locked.

We formulate the time correlation of *f*(*t*) and *g*(*t*) as

(1) $$\frac{1}{T}\int_{t-T}^{t}\;f(\tau )g(\tau )\;\;d\tau$$

where *T* denotes the period of the correlation time window, which is sufficiently longer than the vision frame time.

The main difference between this formulation and the standard theory described in Section 2.1 is that *f*(*t*) takes 1 and 0 instead of 1 and −1. This difference comes from the fact that light brightness cannot be negative. Nevertheless, the PLL system will behave in the same way as the standard one as long as *g*(*t*) has 50% duty ratio, because

(2) $$\frac{1}{T}\int_{t-T}^{t}f(\tau )g(\tau )d\tau$$

(3) $$=\frac{1}{T}\int_{t-T}^{t}\;(\frac{1}{2}f^{\prime }(\tau )+\frac{1}{2})g(\tau )d\tau$$

(4) $$=\frac{1}{2T}\int_{t-T}^{t}f^{\prime }(\tau )g(\tau )d\tau +\frac{1}{2T}\int_{t-T}^{t}g(\tau )d\tau$$

(5) $$=\frac{1}{2T}\int_{t-T}^{t}f^{\prime }(\tau )g(\tau )d\tau$$

where *f*′(*t*) is the square wave with the values 1 and −1. This is a common technique used when correlation of optical signals is computed [12,13].

Unfortunately, we still cannot implement this time correlation computation as it is formulated. Most vision sensors output an image, which is the result of time integration of incident light over the frame time, just frame by frame, and therefore the reference value *f*(*t*) or the product *f*(*t*)*g*(*t*) at any arbitrary time instant is unavailable. However, by considering that *g*(*t*) is a constant during one frame period, we can obtain the time correlation as

(6) $$\frac{1}{T}\int_{t-T}^{t}f(\tau )g(\tau )d\tau =\frac{1}{T}\sum_{i}{\left(-1\right)}^{i-1}F[i]$$

where *i* is the frame number index and *F*(*i*) is the sum of the pixel values obtained within the frame *i*.

This notation assumes that the correlation window length *T* is multiples of the frame time, but we do not stick to this case. Actually, we even do not execute integration over a fixed time window but replace it with discrete-time summation and low-pass filtering. In this case, we have no explicit correlation time windows, but the time constant of the employed low-pass filter plays the corresponding role.

**Figure 6.**
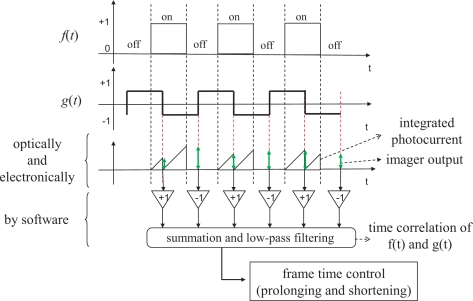
Procedure of the proposed time-correlation computation.

The whole procedure of the proposed time-correlation computation is depicted in Figure 6. The three horizontal axes, from top to bottom, stand for *f*(*t*), *g*(*t*) and an conceptual illustration of the imager output, respectively. The blue and red rectangles on the third axis show integrated photocurrent amount in the pixels in odd and even frames, respectively, and the green vertical arrows show the output from the imager, namely the sum of the pixel values over all the pixels, obtained at the ends of frames, which are proportional to the heights of the blue/red triangles at the corresponding frames. The outputs at odd and even frames are multiplied by 1 and −1, respectively, and fed to the summation and low-pass filtering process.

The resulting correlation is then used to adjust the frame time length of the vision sensor and this process serves as a voltage-controlled oscillator in PLLs. It makes the frame time length equal to a half of the illumination modulation period when the correlation is zero, longer when the correlation is positive, and shorter when negative.

In the literature, image sensor technologies to compute time correlation between incident light brightness and some given signals by introducing multiplication hardware within a pixel have been presented [12,13]. Unlike these prior proposals, we do not need any dedicated pixel structures and thus off-the-shelf image sensors can be used. The reason this is possible is that our application does not require correlation results per pixel but only the sum over all the pixels is needed, which allows us to execute the multiplication outside of the pixel array.

To implement our algorithm, we need to have a means to precisely adjust the frame time length of the vision sensor in real time. Many industrial cameras offer functions to control the shutter by external trigger signals, and then implementing a circuitry to adjust the frame time is easy. In some cameras, we may even have access to built-in functions to control the frame time by software, and such functions can be utilized unless they come with severe processing delays. If there are no means to adjustment the frame time at all, admittedly, our method cannot be applied, and the virtual synchronization will be the only way.

### 2.3. Effect of Background Light

So far, we have assumed that there is no background light, which will not be supposed in most realistic situations. In situations with background light, the incident light accepted by the imager is given by the sum of the light component originated from the modulated illumination and that originated from the other light sources. Note that the former is proportional to the brightness of the modulated illumination and the latter is independent of the modulated illumination.

By remembering that our algorithm always takes the difference of the imager output of successive two frames, we can expect that the background light component will be canceled unless the scene changes too rapidly. This discussion is validated in the following experiments section. When most of the pixels are saturated by background light, the proposed method is, of course, not able to carry out synchronization because the modulated illumination cannot offer any information. Note that this situation, in which most of the pixels are saturated, prohibits almost any kinds of visual information processing.

### 2.4. Effect of Photo Integration Time Shorter than Frame Time

We have also assumed so far that the imager accepts incident light over the whole frame time. In some cases, this is not true and mechanical or electronic shutters are introduced to limit the photo integration time to be shorter.

**Figure 7.**
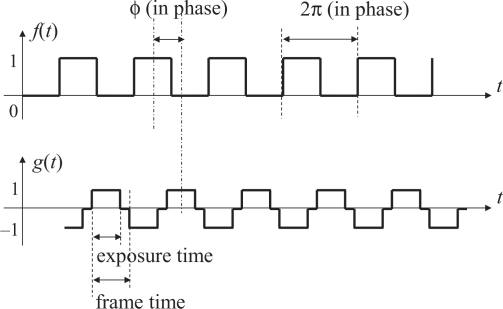
The reference and the output signals in shorter integration time case.

**Figure 8.**
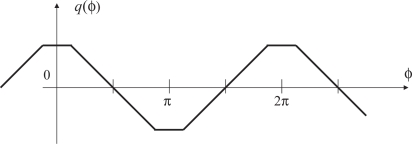
Relation between time correlation and phase difference with non-integration time.

The proposed algorithm will work well even in this shorter integration time case. To illustrate this, the reference and output signals in this case is shown in Figure 7 and the correlation of them is shown in Figure 8. The output signal *g*(*t*) is redefined so that it takes positive or negative values only within the integration time and zero otherwise. The phase difference *ϕ* between *f*(*t*) and *g*(*t*) is redefined so that *ϕ* is zero if the middle time of a period when the modulated illumination is on coincides with the mid time of the integration time of the corresponding frame as shown in Figure 7.

As can be seen from Figure 8, the point *q*(*ϕ*) = *π/*2 is the only stable equilibrium point even in this case. Therefore the proposed algorithm will work without any modification. This discussion is also validated in the simulation and experiment sections later.

It should be emphasized that the modulated, that is, blinking illumination does not disturb the visual observability of scenes. Because the vision sensor gets locked with the *π/*2 phase shift, the sensor operates in such a way that a half of every frame time is always illuminated, and the accumulated incident light within one frame time is always constant as long as the system is locked. Tracking of the locked PLL state can go on simultaneously with visual measurement for applications.

## 3. Simulations

### 3.1. Simulation Setup

This section presents simulated results to analyze the system behavior. The purpose of the simulations is twofold. Firstly, we aim at exploring feasible parameters for the system while evaluating the synchronization performance. We model visual measurement using a high-speed vision sensor with 1,000 Hz frame rate and 64 × 64 pixels, which requires 500 Hz modulated illumination. The frame rate and the number of pixels are decided so that they are equivalent to those of the vision sensor used in the real experiments in the next section.

Secondly, we aim at making sure that the assumption made in the algorithm design, namely that the average brightness of the scene does not change too rapidly, is not too restrictive for the proposed method to be applied to realistic visual measurement. In order to attest this, we use a brightness sequence from a real scene as the envelope of the reference input.

**Figure 9.**
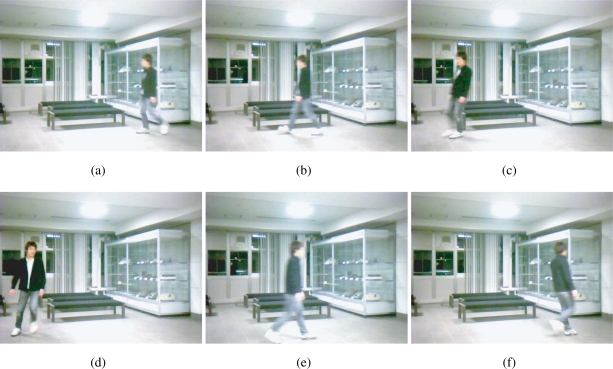
The real scene whose brightness sequence is used as the envelope of the input for simulation, which assumes a surveillance scenario, (a) 0 s, (b) 3.082 s, (c) 7.291 s, (d) 9.689 s, (e) 11.754 s, (f) 13.688 s.

**Figure 10.**
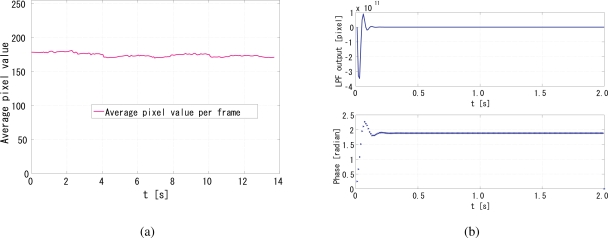
Real-world scene simulation results, (a) average pixel values per frame, (b) output of low pass filter and phase of output signal.

Figure 9 shows snapshots of an indoor scene in which a person is walking around within the field of view of a camera (ELECOM UCAM-DLM130HSV, 640 × 480 pixels, 30 Hz, 8 bits). The average of the pixel values within each frame is computed to obtain the average brightness sequence of the scene *b*(*t*), which is shown in Figure 10 (a). Note that the scene includes a nonnegligible moving region but the average pixel value is almost steady at around 175. The reference signal *f*(*t*) for the simulations is generated from *b*(*t*) and the 500-Hz unity-amplitude square wave *r*(*t*) as *f*(*t*) = *Nb*(*t*)*r*(*t*), where *N* = 64 × 64 is the number of the pixels. This corresponds to a situation that all of the room light of this scene is replaced with the intensity-modulated illumination and no background light exists.

The reference signal *f*(*t*) is injected into the simulator of the proposed algorithm implemented in MATLABR2009b. The discrete-time low-pass filter to give the time correlation *q*[*i*] at frame *i* is implemented as a simple first-order recursive filter

(7) q[i]=k⋅q[i−2]+(1−k)·(F[i−1]−F[i])

computed every two frames, because as shown in Figure 6 the input of low-pass filter is the difference of the integrated pixel values of every two successive frames. The actual value of *k* is explored and analyzed later. It could also be possible to adjust the frame time every frame by, for example, employing a feedback law with a moving window composed of recent two frames, but the simplest one is employed to access the most basic behavior of the system.

Each frame of the simulated vision sensor consists of an integration period and a non-integration period. The integration period is fixed to 0.8 ms, and the length of the non-integration period *τ*_nonint_ is changed in accordance with the time correlation *q*[*i*] every two frames; Specifically, the correlation at frame *i* determines *τ*_nonint_ in the following two frames as

(8) $$\tau_{\text{nonint}}\;[i+1]=\tau_{\text{nonint}}\;[i+2]=\tau_{0}+G\;\cdot q[i]$$

where *τ*_0_ is a constant set to 1 ms, and the gain *G* is a constant whose actual value is explored later. The resolution of adjustment of *τ*_nonint_ is 100 ns, which is the instruction cycle of the system used in the experiment section.

We evaluate the performance of synchronization by the time to convergence and the jitter after convergence. The convergence time, is the time the system takes until the relative phase error of the output signal to the reference becomes stable on the order of 10^−2^ rad. The jitter is evaluated as the standard deviation of the phase of the output signal after the convergence time.

### 3.2. Simulated Results

Figure 10 (b) shows a successful case, where the gain *G* was set to 4 × 10^−9^. The unit of gain is s/pixel, because in Equation (8) *q*[*i*] is in the dimension of the pixel value multiplied by the number of pixels, and the pixels value is dimensionless. The upper figure shows the time correlation for the first two seconds of the simulation, and the lower shows the relative phase of the output signal to the reference for the same period. It can be seen that the system immediately converged to the *π/*2 relative phase and became quite stable. The behavior after 2 s till the simulation end time, which is not shown in this figure, was also stable. This suggests that successful synchronization is possible even with dynamic scenes if the fluctuation of the average brightness is relatively small.

Apparently, the proposed algorithm cannot tolerate too large changes in the average brightness, considering that the appropriate gain depends on the amplitude of the reference signal but we keep the gain constant. Actually, when 5-Hz sinusoidal signals with different amplitudes added to a constant direct-current (DC) component was employed as the reference envelope *b*(*t*), synchronization was successful only as long as the sinusoidal amplitude was within 7.75% of the DC component.

**Figure 11.**
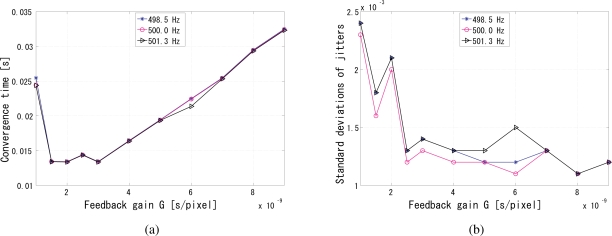
Performance evaluation of the simulation results, (a) convergence time of different gains versus the gain of the input signal, (b) phase jitters versus the gain of the input signal.

The synchronization performance is analyzed in Figure 11 for different gains *G*. In these simulations, we also tested reference signal frequencies slightly different from 500 Hz, because there will be always a small discrepancy between nominal and actual operating frequencies in every clock oscillator.

Figure 11 (a) shows the convergence times for different gains and reference frequencies. Apparently, this indicates that the gain around 3 × 10^−9^ results in the minimum convergence time for this setup. As can be easily foreseen, too small or too large gains make the convergence slow or completely unsuccessful. The discrepancies in the reference frequency did not cause significant changes in the convergence time. The ratio of the upper and lower limits of the gain with which the system converges was approximately twenty, which means the system works well for a reasonably wide gain range.

Figure 11 (b) shows the jitters for different gains and reference frequencies. The jitters were evaluated for the duration between the convergence time and 2 s from the beginning of the measurement. Leastwise, the relative phase between the reference and output is very stable and on the order of 10^−3^ rad within this range of the gain.

**Figure 12.**
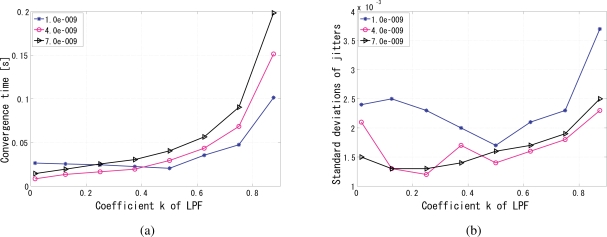
Analysis of the coefficient *k* of the IIR LPF, (a) convergence time versus different coefficient *k* of the LPF, (b) phase jitters versus different coefficient *k* of the LPF.

**Figure 13.**
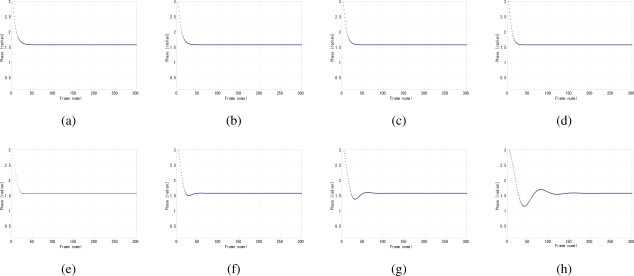
Undershoot phenomena of different coefficient *k* of LPF when *G* = 1.0 × 10^−9^, (a) *k* = 0.0156, (b) *k* = 0.0125, (c) *k* = 0.2500, (d) *k* = 0.3750, (e) *k* = 0.5000, (f) *k* = 0.6250, (g) *k* = 0.7500, (h) *k* = 0.8750.

**Figure 14.**
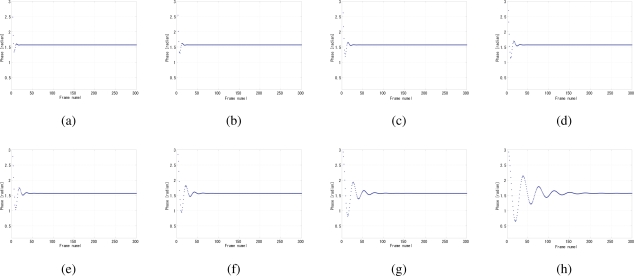
Overshoot phenomena of different coefficient *k* of LPF when *G* = 4.0 × 10^−9^, (a) *k* = 0.0156, (b) *k* = 0.0125, (c) *k* = 0.2500, (d) *k* = 0.3750, (e) *k* = 0.5000, (f) *k* = 0.6250, (g) *k* = 0.7500, (h) *k* = 0.8750.

**Figure 15.**
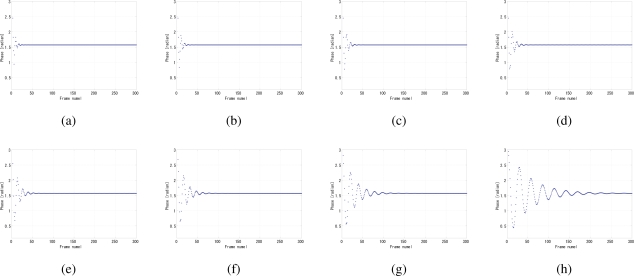
Overshoot phenomena of different coefficient *k* of LPF when *G* = 7.0 × 10^−9^, (a) *k* = 0.0156, (b) *k* = 0.0125, (c) *k* = 0.2500, (d) *k* = 0.3750, (e) *k* = 0.5000, (f) *k* = 0.6250, (g) *k* = 0.7500, (h) *k* = 0.8750.

By analyzing the convergence time and the jitters, the optimal value of the coefficient *k* in Equation (7) can be determined to 0.25, because apparently for a certain gain value such as 4 × 10^−9^ the smaller the *k*, the shorter the convergence time as shown in Figure 12 (a), while at 0.25 the phase jitters are comparably the fewest in Figure 12 (b). The coefficient *k* also has influence on the stability, such as overshoot and undershoot, of system before convergence. Several gain values were tested because the gain and *k* are not independent with each other, as shown in Figure 13, Figure 14, and Figure 15. The larger the *k*, the more severe the oscillation.

## 4. Experiments

### 4.1. Experimental Setup

To demonstrate the proposed method, the algorithm in the same way as the simulations was implemented on a real vision sensor. We employed a high-speed vision system called VCS-IV developed by the authors [14], which captures and processes images in real time at the frame rate up to around 1,000 Hz.

The VCS-IV vision system is equipped with a 64 × 64 pixels CMOS imager called *Digital Vision Chip*, which has capability of pixel-parallel image processing programs on the focal-plane processing element array. This capability is not utilized in the presented experiment except for computation of summation of 6-bits digital pixel values over the array, which is used as *F*[*i*] in the same way as done in simulation. The primary reason we chose this special vision sensor is that it can operate at high frame rate and also its frame time, either the integration period or the non-integration period, can be easily adjusted in a software way without any need of additional control circuitry. Applying the proposed method to any other high-frame rate vision sensor, possibly with larger number of pixels, will be easy if one has a measure to compute the sum of all the pixel values. In the presented experiment, non-integration periods were adjusted just as done in the simulations.

**Figure 16.**
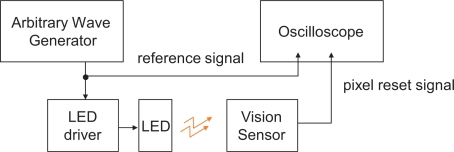
Block diagram of the experimental setup.

Figure 16 shows the block diagram of the experimental setup. The illumination system consists of a Nissin Electronics LDR-90 LED array and an LPR-30W-D power supply system, which are driven by the reference square-wave signal from a Tektronics AFG3102 arbitrary wave generator.

The operation of the vision system was measured by observing the pixel reset signal of the imager, whose positive edge corresponds to the beginning of an integration period, by a Tektronics TDS3034 oscilloscope. If the operation of the vision system is locked to the illumination, synchronized waveforms of the pixel reset and the reference signal will be observed in the oscilloscope.

**Figure 17.**
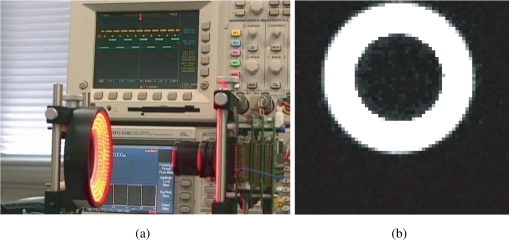
Experimental scene and image, (a) successful experiment, (b) an obtained frame of vision chip.

The LED directly shed light on the vision system with an imaging optics in a normal laboratory environment as shown in Figure 17 (a). The illuminance measured in front of the imaging optics when the LED light is off was 187 lx, and was 3460 lx when the LED is on without intensity modulation. An image taken by the vision system when the LED is on without modulation is shown in Figure 17 (b), which shows that the pixels receiving light from the LED are almost saturated. Note that the imager used here has considerably low sensitivity and is noisy, and the background texture is almost unobservable. The average pixel value over both of the illuminated and unilluminated pixels during a 50 frames sequence was 18.4 with 0.053 standard deviation, while it was 0.8 with 0.036 standard deviation when the LED is off.

### 4.2. Experimental Results

**Figure 18.**
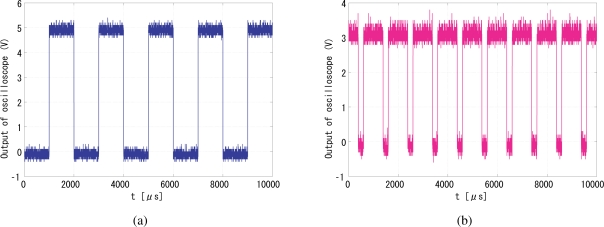
Successful experimental results, (a) input signal, (b) output signal.

Figure 18 shows snapshots of the 500 Hz square-wave signal to drive the LED and the output (pixel reset) signal that successfully locked to the illumination reference, where the gain was set to 16. The output signal got synchronized to the reference signal with *π/*2 relative phase shift and twice the frequency. The running system is shown in the attached video clip.

As demonstrated by the simulations, changing the reference frequency does not vitally affect the synchronization stability. Actually, the reference signal frequency allowing stable synchronization ranged from 343 Hz to 606 Hz in the presented condition while the central frame rate of the vision system is fixed to 1,000 Hz. However, the larger the frequency discrepancy was, the larger the observed steady-state phase error from the *π/*2 shift was. This is explained by the fact that non-zero time correlation must be produced to fix the vision frame rate away from its central value. This will be compensated by a more sophisticated PLL design.

To attest the robustness against background light changes, an incandescent electric torch illuminating the vision system was moved randomly in front of the vision system. It clearly shows in the video that the torch almost did not affect the stability of the system.

**Figure 19.**
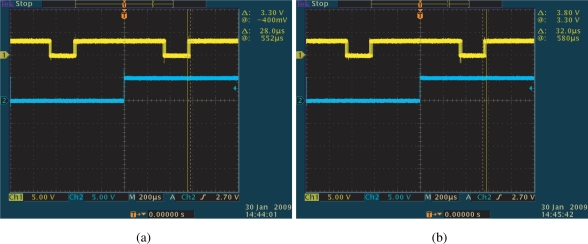
Jitters comparison concerning the torch illumination, (a) jitters without the incandescent torch disturbance, (b) jitters with the incandescent torch disturbance.

The peak-to-peak jitter of the output signal measured by the oscilloscope was around 28 *μ*s in the normal condition shown in Figure 19 (a), and 32 *μ*s with the randomly moving torch shown in Figure 19 (b), which is 1.5% of the reference period and thus 0.1-rad phase error. The 1.5% jitter is considerably large compared to the simulated results. This result can be justified considering that the employed imager suffers from severe noise.

## 5. Conclusions

An illumination-based synchronization method based on PLL for high-speed vision sensors has been described. Experimental results showed that the operation of sensors can be successfully locked to an LED illumination signal as long as the gain parameter was carefully chosen to fit the brightness of illumination. It has been proved that the current algorithm can tolerate brightness fluctuations of background light, as well as small extent of changes of modulated illumination signal. This dependency should be removed in future work by introducing a procedure to normalize the input signal amplitudes.
